# EGCG Attenuates Uric Acid-Induced Inflammatory and Oxidative Stress Responses by Medicating the NOTCH Pathway

**DOI:** 10.1155/2015/214836

**Published:** 2015-10-11

**Authors:** Hua Xie, Jianqin Sun, Yanqiu Chen, Min Zong, Shijie Li, Yan Wang

**Affiliations:** Nutrition Department, Huadong Hospital Affiliated to Fudan University, Shanghai 200040, China

## Abstract

*Background*. The aim of this study is to investigate whether (-)-epigallocatechin-3-gallate (EGCG) can prevent the UA-induced inflammatory effect of human umbilical vein endothelial cells (HUVEC) and the involved mechanisms in vitro. *Methods*. HUVEC were subjected to uric acid (UA) with or without EGCG treatment. RT-PCR and western blots were performed to determine the level of inflammation marker. The antioxidant activity was evaluated by measuring scavenged reactive oxygen species (ROS). Functional studies of the role of Notch-1 in HUVEC lines were performed using RNA interference analyses. *Results*. UA significantly increased the expressions of IL-6, ICAM-1, TNF-*α*, and MCP-1 and the production of ROS in HUVEC. Meanwhile, the expression of Notch-1 and its downstream effects significantly increased. Using siRNA, inhibition of Notch-1 signaling significantly impeded the expressions of inflammatory cytokines under UA treatment. Interestingly, EGCG suppressed the expressions of inflammatory cytokines and the generation of ROS. Western blot analysis of Notch-1 showed that EGCG significantly decreased the expressions of inflammatory cytokines through Notch-1 signaling pathways. *Conclusions*. In summary, our findings indicated that Notch-1 plays an important role in the UA-induced inflammatory response, and the downregulation of Notch-1 by EGCG could be an effective approach to decrease inflammation and oxidative stress induced by UA.

## 1. Introduction

Hyperuricemia has been reported to increase the risk of gout, renal diseases, and cardiovascular diseases, such as hypertension and atherosclerosis [1]. Though its etiology is unclear, hyperuricemia can be secondary either to an exaggerated production of UA that follows high cellular metabolic conditions or to a low renal excretion in patients with renal function impairment [2, 3]. The tissue accumulation of UA leads to the formation of crystalline deposits, which triggers endothelial dysfunction and the outset of an inflammatory response [2, 4]. Inflammation is known to play a role in the progression of various disorders such as hypertension, atherosclerosis, and diabetes [5–11]. Useful topical treatment for hyperuricemia is limited.

Emerging evidence confirms that Notch signaling play a key role in inflammatory response involved in the pathogenesis of cardiovascular diseases [12–14]. Notch ligands and receptors have been reported to increase in damaged myocardial and vessels [15–17]. However, little is known about the association between UA levels and the expression of Notch-1.

EGCG is a flavanol derived from green tea extracts (GTE) and recently has been explored in disease therapy because of its antioxidant, anti-inflammatory, and anticancer effects [18–21]. Previous studies have shown that EGCG-mediated cardioprotection against the H_2_O_2_-induced oxidative stress by Akt/GSK-3*β*/caveolin signaling in vitro and in vivo [22]. In addition, EGCG inhibits cytochrome c release and activation of pro-caspase-3 to improve cigarette smoke-induced myocardial dysfunction [23]. EGCG increases production of NO via a phosphatidylinositol (PI) 3-kinase/Akt-medicating endothelial nitric oxide synthase (eNOS) in vascular endothelial cells and enhance insulin sensitivity to improve endothelial function [24, 25]. Recent study has shown that EGCG regulates Notch signaling to inhibit the proliferation of colorectal cancer cells [26]. However, the role of EGCG on the effect of hyperuricemia has been unresolved.

In this study, we evaluated that treatment of HUVEC with UA activated inflammatory response and oxidative stress. Next, we examined the potential mechanism which revealed that Notch-1 was essential for UA-induced inflammatory response and oxidative stress. Further experiments indicated that EGCG mediated vascular endothelial cells protection in an UA-induced inflammatory response and oxidative stress through Notch pathway, at least partly.

## 2. Materials and Methods

### 2.1. Materials

UA and EGCG were purchased from Sigma-Aldrich (St. Louis, MO, USA). EGCG stock solution was prepared in sterile double distilled water at 20 mM. UA was dissolved in deionized water and filtered and was free of crystals (by polarizing microscopy).

### 2.2. Cell Culture and Cell Transfection

The human umbilical vein endothelial cells (HUVEC) were purchased from ATCC (Rockville, MD, USA). HUVEC were grown in EGM-2 Bullet kit (Lonza, Basel, Switzerland) containing penicillin G (100 Units/mL; Sigma-Aldrich) and streptomycin sulfate (100 *μ*g/mL; Sigma-Aldrich) at 37°C in a humidified atmosphere of 5% CO_2_. HUVEC were cultured with UA (8 mg/dL) for another 24 hours after pretreating with or without EGCG (20*μ*M).

Small interference RNA (siRNA) targeting Notch-1 (numbered 1–3) and a scrambled siRNA were designed and purchased from GenePharma Co., Ltd. (Shanghai, China). The effective complementary (upper) sequences in the two RNA duplexes were 5′-GCACGCGGAUUAAUUUGCAdTdT-3′ and 5′-UGCAAAUUAAUCCGCGUGCdTdT-3′. The full cDNA of Notch-1 was cloned in the pcDNA3.1 vector (Invitrogen) and was confirmed by DNA sequencing. For transient silencing, 5 × 10^5^/mL cells were seeded onto 60 mm dish and transfected with Notch-1 siRNA (50 nmol/L) using Lipofectamine 2000 (Invitrogen Corp., Carlsbad, CA, USA) according to manufacturer's protocol. The medium was replaced with complete medium and incubated at 37°C for 48 hours before further analysis. The transfection efficacy was evaluated by western blot.

### 2.3. Measurement of Intracellular ROS Production by Fluorescence Spectrophotometry

To determine whether the ROS levels of HUVEC are affected by the presence of UA or EGCG, a chemiluminescence assay was assessed using CellROX Green Reagent (Life technologies). The examined HUVEC were seeded onto 24-well plates and treated with various processing. CellROX Green Reagent was incubated to each well at a concentration of 10 *μ*mol/L and mixed vigorously for 1 hour at 37°C. Fluorescence of CellROX was measured with a laser scanning confocal microscope (Leica, TCS SP2, Bensheim, Germany) at 488 nm of excitation wavelength and 510 nm of emission filter. The semiquantified ROS was expressed as relative fluorescent units (RFU) and all experiments were performed in duplicate and repeated three times.

### 2.4. RNA Extraction and Quantitative Real Time PCR (qRT-PCR)

Total RNA from cells was extracted using Trizol (Invitrogen Corp) following the manufacturer's protocol. cDNA was synthesized using 500 ng of total RNA by Transcriptor First Strand cDNA Synthesis Kit (Roche, Mannheim, Germany). The resulting first-strand cDNA was amplified in a final volume of 20 *μ*L containing 10 pmol of each primer by One Step SYBR PrimeScript RT-PCR Kit II (TaKaRa, Shuzo, Japan). The oligonucleotide primers that were used for the PCR amplifications were synthesized by Shanghai Sangon Technologies, Inc. (Sangon, China) and are listed as follows: forward primer: Hes1 forward primer: 5′-CCTGTCATCCCCGTCTACAC-3′, reverse primer: 5′-CACATGGAGTCCGCCGTAA-3′; MCP-1, forward primer: 5′-CAGCCAGATGCAATCAATGCC-3′, reverse primer: 5′-TGGAATCCTGAACCCACTTCT-3′; NF-*κ*B, forward primer: 5′-GAAGCACGAATGACAGAGGC-3′, reverse primer: 5′-GCTTGGCGGATTAGCTCTTTT-3′; ICAM-1, forward primer: 5′-TTGGGCATAGAGACCCCGTT-3′, reverse primer: 5′-TGGAATCCTGAACCCACTTCT-3′; TNF-*α*, forward primer: 5′-CCTCTCTCTAATCAGCCCTCTG-3′, reverse primer: 5′-GAGGACCTGGGAGTAGATGAG-3′; GAPDH, forward primer: 5′-ACAACTTTGGTATCGTGGAAGG-3′, reverse primer: 5′-GCCATCACGCCACAGTTTC-3′. A melting curve analysis was performed for each of the primers used, and each showed a single peak indicating the specificity of each of the primers tested. All values were calculated using the delta Ct method and expressed as the change relative to the expression of glyceraldehyde 3-phosphate dehydrogenase (GAPDH). The amplification conditions were 40 cycles of denaturation at 94°C for 1 min, annealing at 60°C for 30 s.

### 2.5. Western Blot Analysis

Cell lysates were rinsed with ice-cold PBS and prepared using lysis buffer with 1% Triton, 1% Nonidet P-40, 0.1% sodium dodecyl sulfate (SDS), and 1% deoxycholate and protease inhibitors cocktail (Roche). Protein concentration was determined by Bradford protein assay kit (Bio-Rad) and equal amount of protein was separated by 12% SDS-PAGE using Bio-Rad apparatus. The membranes were blocked for 2 hours in 5% skim milk at room temperate after protein was transferred to PVDF membrane (Millipore, Billerica, MA, USA). The proteins were incubated with the following primary antibodies: IL-6 and TNF-*α* (Merck Millipore, Bedford, MA, USA), ICAM-1, Hes5 and MCP-1 (Abcam, Cambridge, Mass, USA), Hes1 (OriGene Technologies, Rockville, MD, USA), Hey1 and Hey2 (Proteintech Group, Chicago, IL, USA), and phospho-p65 (CST, Chicago, IL, USA). Anti-GAPDH antibody (Bioworld, Nanjing, China) was used as the loading control in all western blots. The secondary antibodies, anti-mouse and anti-rabbit IgGs conjugated to horseradish peroxidase, were obtained from Kangchen (Shanghai, China). Finally, protein bands were visualized using Supersignal West Femto Substrate (Pierce, Rockford, IL, USA).

### 2.6. Statistical Analysis

The results were presented as means ± SD. The statistical significance of differential findings was statistically evaluated using GraphPad StatMate software (GraphPad Software, Inc., San Diego, CA). Statistical significance for comparisons between groups was determined using Student's paired two-tailed* t*-test or analysis of variance (ANOVA). A *P* value of <0.05 was considered statistically significant.

## 3. Results and Discussion

### 3.1. Results

#### 3.1.1. High Uric Acid Level Induces Inflammatory Responses and Oxidative Stress in HUVEC

**Figure 1:**
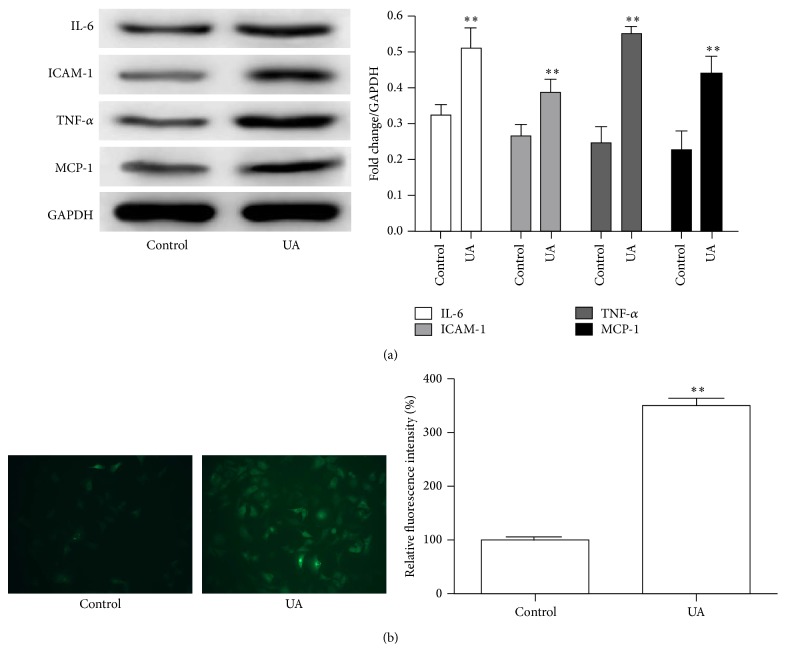
Effect of UA on the expression of IL-6, ICAM-1, MCP-1, and TNF-*α* and the ROS production in HUVEC. (a) Western blot analysis for IL-6, ICAM-1, MCP-1, and TNF-*α* in HUVEC after incubation with UA (8 mg/dL). (b) Representative images showing that intracellular ROS production was detected using CellROX Green Reagent. Histogram illustrating ROS production showed a different response compared with UA. ^*∗∗*^ *P* < 0.01.

Previous studies indicated that hyperuricemia was associated with hypertension, systemic inflammation, and cardiovascular disease mediated by endothelial dysfunction and pathologic vascular remodeling [27]. To investigate the effects of UA on HUVEC, we examined the expression of inflammatory chemokines by western blot. UA remarkably increased the expression of IL-6, ICAM-1, MCP-1, and TNF-*α* at a concentration of 8 mg/dL (Figure 1(a)) in HUVEC. Recent studies have shown that inflammatory response induced the generation of reactive oxygen species (ROS) in an NADPH oxidase-dependent manner in endothelial cells [28]. Then we further investigated the effects of UA on the ROS production. As shown in Figure 1(b), UA significantly increased the ROS production in HUVEC (3.28 ± 0.34-fold, *P* = 0.010).

#### 3.1.2. High Uric Acid Level Upregulates Notch-1 Expression and Activates NOTCH Signaling

**Figure 2:**
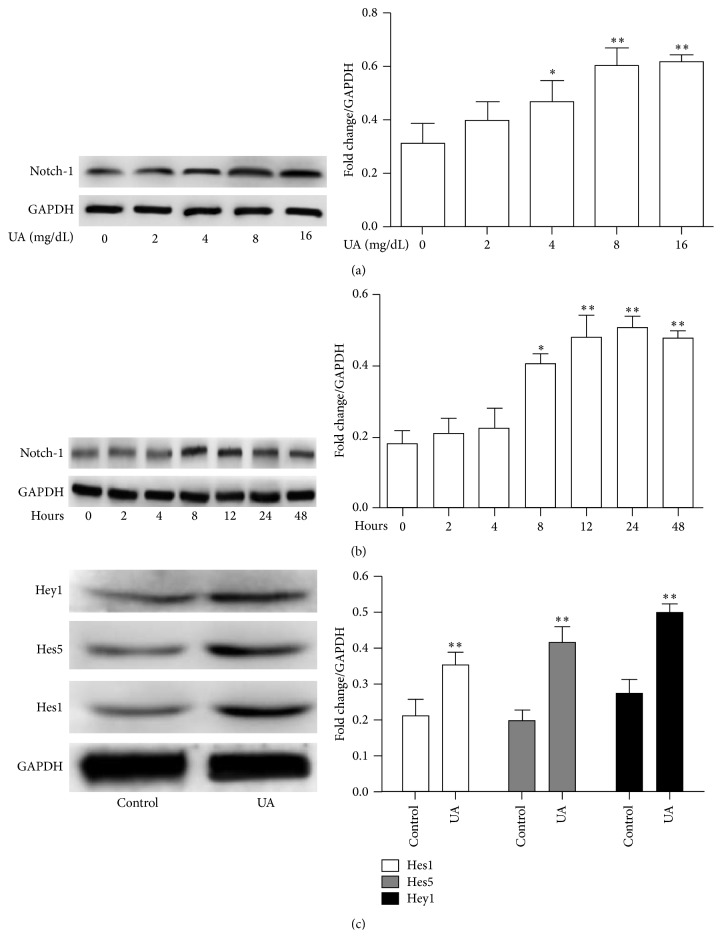
Dose- (a) and time-dependent (b) effect of UA on Notch1 expression in HUVEC. (a) UA enhanced the expression of Notch1 at concentrations of 8 mg/dL or higher compared with control. (b) UA-induced expression of Notch1 peaked at 8 hours and remained elevated at 48 hours. (c) Western blot analysis of Hes1, Hes5, and Hey1 protein expressions in HUVEC treated with UA (8 mg/dL) for 8 hours.

Upregulation of Notch-1 played important roles in inflammatory reaction [13, 29]. To investigate whether Notch-1 is regulated by UA, we examined Notch-1 expression by introducing UA. As presented in Figure 2(a), UA induced intracellular Notch-1 levels in a dose-dependent manner, and maximal stimulation was achieved at 8 mg/dL (*P* < 0.05). The expression of Notch-1 induced by UA was also time-dependent, being significantly higher than that of control by 8 hours, peaking after 24 hours of stimulation (*P* < 0.05; Figure 2(b)). To further examine the activation of NOTCH signaling after UA treatment, Hes1, Hes5, Hey1, and Hey2 protein expression were evaluated by western blot analysis. As shown in Figure 2(c), UA increases the protein levels of Hes1, Hes5, and Hey1, but not of Hey2 in HUVEC (data not shown). These data suggested that NOTCH signaling pathway was involved in damage induced by UA.

#### 3.1.3. NOTCH Silencing Restricts UA-Induced Inflammatory Responses and Oxidative Stress

**Figure 3:**
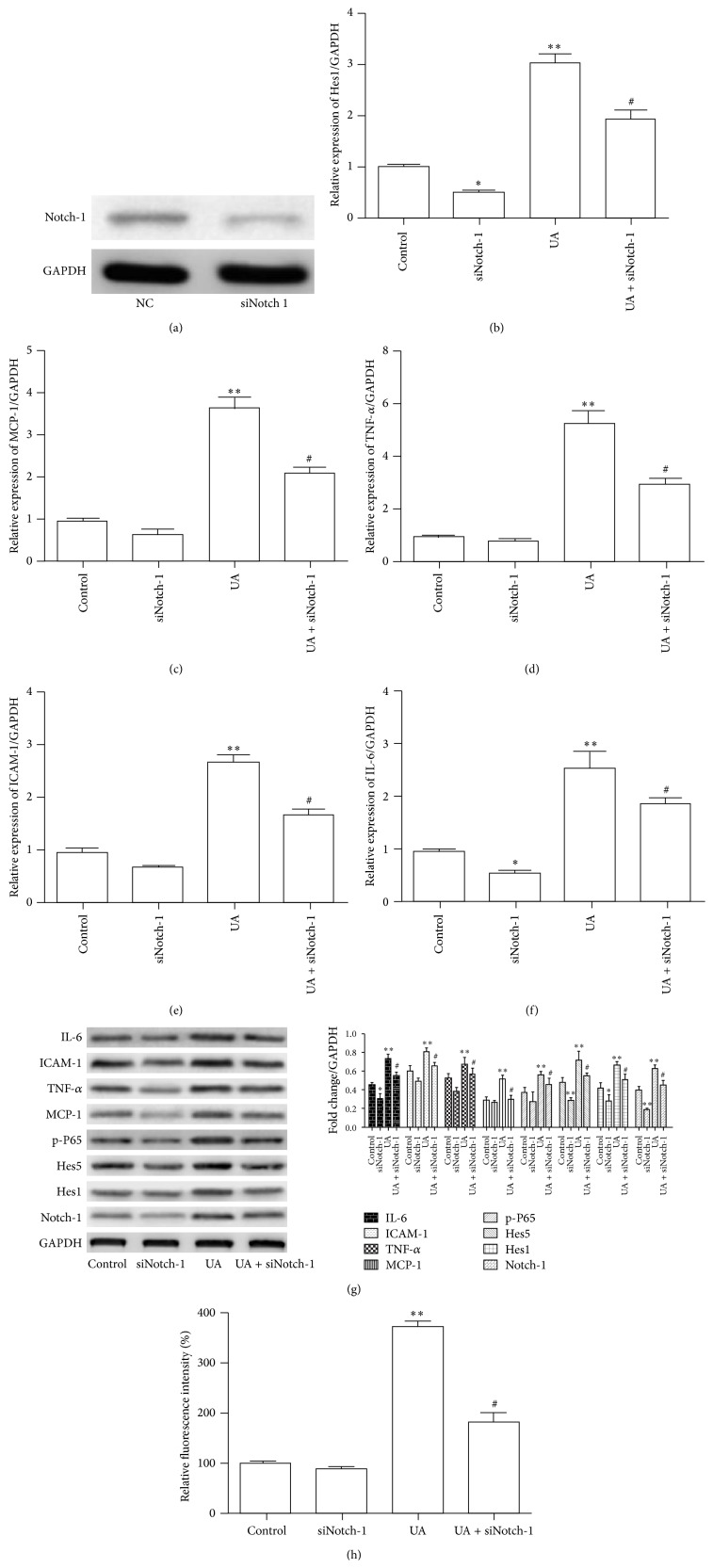
Notch signaling pathway was involved in UA-induced inflammatory reaction and oxidative stress. (a) The expression of Notch1 was confirmed by western blotting analysis in HUVEC transfected with Notch1 siRNA. (b–f) The Hes1, MCP-1, TNF-*α*, ICAM-1, and NF-*κ*B mRNA were detected qRT-PCR in HUVEC with treatment as indicated. (g) Immunoblot analysis showing effects of UA with or without Notch1 inhibition by siRNA on the expression of Notch-1, IL-6, MCP-1, ICAM-1, TNF-*α*, Hes1, Hes5, and p-P65 in HUVEC. (h) Quantitative analysis of intracellular ROS production for the HUVEC with the indicated treatment. ^*∗*^ *P* < 0.05, ^*∗∗*^ *P* < 0.01.

We had previously reported that UA led to increased mRNA expression of inflammatory chemokines, such as MCP-1, ICAM-1, P65, and TNF-*α*. Next, to further investigate whether activation of the NOTCH signaling pathway was involved in UA-induced inflammatory reaction, we transfected cells with Notch-1-specific siRNA and western blot analysis confirmed effective knockdown of Notch-1 (Figure 3(a)). As expected, downregulation by siRNA decreased the mRNA levels of IL-6, MCP-1, ICAM-1, P65, and TNF-*α* (Figures 3(b)–3(f)). To confirm these results, we also analyzed the protein levels and found that downregulation of Notch-1 expression resulted in decreased expression of p-P65, Hes1, IL-6, MCP-1, ICAM-1, and TNF-*α*, which was induced by UA (Figure 3(g)). Furthermore, we examined the ROS production when Notch-1 knockdown and our results showed that Notch-1 silencing significantly decreased the ROS production compared to control in HUVEC (Figure 3(h)). These results clearly indicated that Notch-1 plays a crucial role in UA-induced inflammatory reaction and oxidative stress.

#### 3.1.4. EGCG Attenuate the Effect of UA

**Figure 4:**
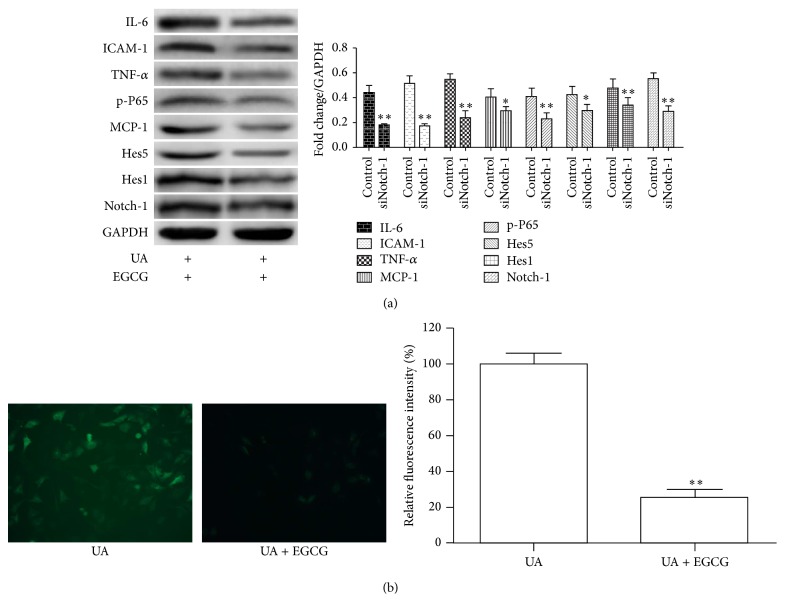
A cell model illustrating cardioprotection of EGCG on UA-induced inflammatory reaction and oxidative stress in HUVEC. (a) Immunoblot analysis reporting the protein levels of Notch1, IL-6, MCP-1, ICAM-1, TNF-*α*, Hes1, Hes5, and p-P65 in whole cell lysates of HUVEC with treatment as indicated. (b) Representative fields of intracellular ROS production in HUVEC treated with UA with or without EGCG. Corresponding densitometric analysis showed the relative ROS production. ^*∗∗*^ *P* < 0.01.

Studies have shown that the EGCG plays an important role in antioxidant and anti-inflammatory effects in multiple physiological processes [19, 20, 25]. To assess the biological activities of EGCG on HUVEC damaged by UA, we detected the protein levels of IL-6, MCP-1, ICAM-1, TNF-*α*, Notch-1, Hes1, Hes5, and Hey1 and the results showed that EGCG could significantly inhibit the expression of inflammatory chemokines (Figure 4(a)). Additionally, consistent with previous results, we found that preincubation with EGCG significantly suppresses ROS production induced by UA. Thus, these data indicated that EGCG effectively reduced inflammatory responses and oxidative stress and might thus help in patients with hyperuricemia.

#### 3.1.5. Overexpression of Notch-1 Reduced Protection of EGCG in HUVEC

**Figure 5:**
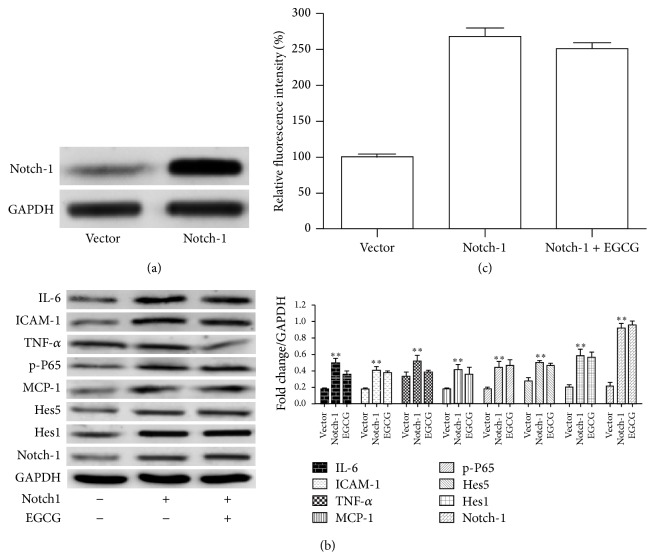
Effects of EGCG on UA-medicated Notch inflammatory signaling pathway. (a) Western blotting analysis of Notch1 was performed to assess the Notch1 transfection in HUVEC. (b) Representative western blot images of proteins of the Notch pathway and inflammatory chemokines detected in whole-cell lysates with antibodies indicated. HUVEC were transfected with Notch1 cDNA with or without EGCG in medium containing UA (mg/dL). (c) Quantitative analysis of intracellular ROS production for the HUVEC with the indicated treatment.

To further investigate whether EGCG regulates Notch-1-mediated inflammatory responses and oxidative stress, we transfected cells with pcDNA3.1-Notch-1 plasmid. Western blot analysis showed that the protein level of Notch-1 expression was significantly increased in HUVEC compared to control vector transfected cells (Figure 5(a)). Moreover, to prove that the overexpression of Notch-1 could inhibit the EGCG decreased downstream protein, we performed western blotting analysis. As we expected, the protein level of IL-6, MCP-1, ICAM-1, TNF-*α*, Notch-1, Hes1, and Hes5 was reversed after Notch-1 overexpression (Figure 5(b)). Consistent with above results, there was no significant difference in HUVEC transfected with Notch-1 with or without EGCG (Figure 5(c)). In summary, our results indicated that EGCG regulated UA-induced cell damage partly through mediating NOTCH signaling pathway.

### 3.2. Discussion

Previously, we have shown the cardioprotection of EGCG against inflammatory lesions induced by UA via the NF-*κ*B signaling pathway. In the current study, we further investigated the effects of UA and EGCG on inflammatory responses and oxidative stress in HUVEC. We found that Notch-1 was involved in UA-induced inflammatory responses and oxidative stress. Moreover, we found that pretreatment with EGCG significantly decreased the expression of Notch-1 and its downstream genes. When we were writing this paper, Jatuworapruk et al. reported that EGCG could modestly lower serum uric acid (SUA) level and significantly elevated serum antioxidant capacity in healthy individuals [30]. These results, for the first time, provided a mechanistic link between the high levels of UA and the antioxidant and anti-inflammatory effects of EGCG.

Recent studies have shown that hyperuricemia characterized by high serum uric acid level was associated with hypertension, gout, systemic inflammation, and cardiovascular disease mediated because of endothelial dysfunction and pathologic vascular remodeling [1, 2, 13, 27]. In the present study, treating the HUVEC with UA, we found that UA significantly increased the inflammatory cytokines and ROS production. Recent evidence has demonstrated that NOTCH signaling played an important role during inflammation in cardiovascular disorders [31]. However, the roles of NOTCH signaling in HUVEC were not well understood. Then we focused on the effect of UA on the Notch-1 expression. Our findings indicated that UA activated Notch-1 in a dose- and time-dependent manner. NOTCH target genes Hes1, Hes5, Hey1, and Hey2 were further determined at protein level and the expression of Hes1, Hes5, and Hey1, but not Hey2, was significantly increased. To further assess UA-induced inflammatory responses and oxidative stress involved in NOTCH signaling, we knockdown Notch-1 expression by siRNA. Inhibition of Notch-1 expression remarkably decreased the downstream genes of Notch-1 and inflammatory chemokines both at mRNA and protein levels. Our results suggested that the effects of UA in HUVEC resulted in inflammatory responses and oxidative stress involved in altering the expression of Notch-1 and its downstream genes.

Previous study showed that EGCG has potent properties of antioxidant and radical-scavenger [19, 24, 25]. Chen et al. have reported that EGCG could protect H9c2 rat cardiomyoblasts against H_2_O_2_-induced oxidative stress via the Akt/GSK-3*β*/*β*-catenin and caveolae signaling [32]. It reminds us whether EGCG could reduce the expression of inflammatory chemokines and generation of ROS. As we expected, EGCG pretreatment reduced the expression of inflammatory chemokines and prevented the release of ROS. Recent study has shown that EGCG inhibited the Notch signaling to suppress the proliferation of colorectal cancer cells [26], in order to determine whether the molecular mechanism by which EGCG inhibited inflammatory responses and oxidative stress of HUVEC cell involved in NOTCH signaling proteins. Supporting this, overexpression of Notch-1 attenuated the effects of EGCG and enhanced the expression of inflammatory chemokines and the release of ROS. The results of this study could be useful for patient with hyperuricemia in order to improve the life quality.

## 4. Conclusions

In summary, our current findings suggested that EGCG inhibited the UA-induced inflammatory responses and oxidative stress through Notch-1 medicating inflammatory chemokines and ROS production. However, further investigations are required to determine the real effect in vivo.
